# A study on metabolic differences between infectious and non-infectious stones in patients with urine culture positive for *Proteus mirabilis*

**DOI:** 10.1186/s12894-026-02116-2

**Published:** 2026-03-23

**Authors:** Endi Zhang, Zhichao Chi, Xuming Zhang, Jianxing Li, Chaoyue Ji, Weiguo Hu

**Affiliations:** Department of Urology, Beijing Tsinghua Changgung Hospital, School of Clinical Medicine, Tsinghua Medicine, Tsinghua University, Beijing, 102218 China

**Keywords:** Urolithiasis, Infectious stones, *Proteus mirabilis*, 24-h urine, Metabolic analysis

## Abstract

**Objective:**

To compare the clinical characteristics of infectious and non-infectious stones in patients with urinary tract infections caused by *Proteus mirabilis* and to explore associated influencing factors.

**Methods:**

Clinical data from 93 patients with upper urinary tract stones and concomitant *Proteus mirabilis* urinary tract infection treated between 2015 and 2021 were retrospectively analyzed. Patients were classified according to postoperative stone composition. Demographic characteristics and preoperative laboratory parameters were compared between groups, and multivariate logistic regression analysis was performed to identify factors associated with infectious stones.

**Results:**

Of the 93 patients included, 70 (75.3%) had infectious stones and 23 (24.7%) had non-infectious stones. Patients in the infectious stone group were younger and had a lower body mass index (BMI) than those in the non-infectious stone group (*P* < 0.05). The 24-h urine volume was significantly lower in the non-infectious stone group (*P* < 0.05). Multivariate analysis showed that older age, higher BMI, and lower urine volume were significantly negatively associated with the presence of infectious stones, whereas diabetes mellitus was significantly associated with infectious stones.

**Conclusion:**

Patients with non-infectious stones should be advised to increase fluid intake and manage body weight. In contrast, patients with infectious stones, particularly those with concomitant diabetes mellitus, require strict glycemic control and active anti-infective treatment to reduce the risk of stone formation.

## Introduction

Urolithiasis is one of the most common urological disorders worldwide and has a relatively high overall prevalence. Previous studies have reported that more than 15% of the global population is affected by urolithiasis [1]. In recent years, both the incidence and prevalence of urolithiasis have shown an increasing trend among males and females [2]. With the rising prevalence of urolithiasis, the associated burden on global healthcare systems continues to increase [3, 4]. It is estimated that the annual expenditure on stone management in the United States alone reaches approximately $2 billion, and this cost continues to rise [5]. These trends highlight the growing urgency for effective strategies for the treatment and prevention of urolithiasis.

Infection stones represent a specific subtype of urinary tract calculi, accounting for approximately 10–15% of all urinary stones [6]. Their formation is closely associated with infections caused by urease-producing bacteria and is characterized by rapid stone growth and a high risk of recurrence. In some cases, staghorn calculi may develop within a relatively short period. Without timely intervention, infection stones can lead to serious complications, including recurrent urinary tract infections, renal impairment, and even progression to urosepsis [7, 8]. The primary components of infection stones are magnesium ammonium phosphate hexahydrate (struvite) and carbonate apatite, with struvite being the predominant constituent [9]. Urine culture findings frequently demonstrate infection with urease-producing bacteria, among which *Proteus mirabilis* is the most commonly identified pathogen [10]. These bacteria hydrolyze urea into ammonium ions, resulting in alkaline urine. Changes in urinary pH influence stone composition: carbonate apatite begins to crystallize at a urine pH of 6.8, whereas struvite precipitates only when the pH reaches 7.2 or higher [11].

Although previous studies have investigated the microbial distribution and metabolic characteristics of infection stones, limited data are available regarding the biochemical and metabolic differences between patients forming different stone types under infection with the same bacterial species. Therefore, this study retrospectively analyzed the clinical data of 93 patients hospitalized at our center between January 2015 and December 2021 who had upper urinary tract stones and a positive urine culture for *Proteus mirabilis*. The aim was to compare general clinical characteristics and blood/urine biochemical parameters between patients with non-infection stones and those with infection stones, and to identify factors associated with the formation of infection-related upper urinary tract stones. The findings of this study are expected to provide useful reference information for the prevention and management of upper urinary tract infection stones associated with *Proteus mirabilis* infection.

## Materials and methods

### Study patients

This study was approved by the Ethics Committee of Beijing Tsinghua Changgung Hospital, Tsinghua University, and was conducted in strict accordance with the ethical principles of the Declaration of Helsinki (1964) and its subsequent amendments. Written informed consent was obtained from all participants. A total of 93 patients with upper urinary tract stones and a preoperatively confirmed urine culture positive for *Proteus mirabilis* were included in this study. Among these patients, 21 were male (22.6%) and 72 were female (77.4%), yielding a male-to-female ratio of 1:3.4. The patients ranged in age from 33 to 85 years, with a mean age of 56.0 ± 11.9 years.

Inclusion criteria were as follows: (1) age ≥ 18 years; (2) preoperative urine culture confirming infection with *Proteus mirabilis*; (3) diagnosis of upper urinary tract stones based on imaging examinations; and (4) surgical treatment with available postoperative stone composition analysis. Exclusion criteria were as follows: (1) incomplete clinical or laboratory data; (2) co-infection with other pathogenic bacteria; (3) presence of predisposing metabolic disorders (e.g., renal tubular acidosis, primary hyperoxaluria) or a history of surgical procedures recognized as iatrogenic causes of stone formation; (4) pregnancy or lactation; and (5) administration of antibiotics within 7 days prior to admission.

### Diagnostic methods

Baseline data, including sex, age, blood pressure, height, weight, and medical history (hypertension and diabetes), were collected. Laboratory assessments included serum biochemical parameters, 24-h urine metabolic profiles, and stone composition analysis. Relevant imaging data were also reviewed. The diagnostic criteria for comorbidities were defined as follows. Hypertension was diagnosed according to the 2020 International Society of Hypertension Guidelines [12]. Diabetes mellitus was diagnosed based on the 2021 American Diabetes Association Standards of Medical Care [13]. Hyperuricemia was defined as a serum uric acid level > 420 μmol/L, in accordance with the 2020 American College of Rheumatology Gout Management Guidelines [14]. Elevated parathyroid hormone (PTH) levels were defined as ≥ 65 ng/L, based on the reference range of our center’s laboratory. Abnormalities in 24-h urine metabolic parameters were defined according to the reference values of our institution: urine volume < 2000 mL/24 h was considered low urine volume; urinary calcium > 7.5 mmol/24 h was defined as hypercalciuria; urinary sodium > 220 mmol/24 h as hypernatriuria; urinary phosphate > 42.0 mmol/24 h as hyperphosphaturia; and urinary uric acid > 4430 μmol/24 h as hyperuricosuria. Based on Fourier-transform infrared spectroscopy (FTIR) analysis, patients with upper urinary tract stones containing ≥ 50% infectious stone components (magnesium ammonium phosphate hexahydrate and/or carbonate apatite) were classified into the infection stone group. The remaining patients were assigned to the non-infection stone group. Differences in baseline characteristics, comorbidities, and blood/urine laboratory parameters between the two groups were compared. In addition, potential risk factors associated with the formation of infection stones in patients with *Proteus mirabilis*-positive urine cultures were explored.

### Statistical analysis

Statistical analyses were performed using SPSS version 27.0. Data distribution normality was assessed prior to analysis. Continuous variables with a normal distribution are presented as mean ± standard deviation (SD) and were compared between groups using the independent-samples t-test. Continuous variables with a non-normal distribution are expressed as median (P25-P75) and were compared using the Mann–Whitney U test. Categorical variables are presented as number (percentage) [n (%)], and group comparisons were conducted using the Chi-square test or Fisher’s exact test, as appropriate. Multivariate logistic regression analysis was performed to identify independent risk factors associated with the formation of upper urinary tract infection stones in patients with a *Proteus mirabilis*-positive urine culture. A P value < 0.05 was considered statistically significant.

## Results

### Baseline characteristics of patients in the non-infection and infection stone groups

Among the 93 patients with upper urinary tract stones and a urine culture positive for *Proteus mirabilis* (Table 1), 23 patients (24.7%) were classified into the non-infection stone group, while 70 patients (75.3%) were assigned to the infection stone group. The mean age of patients in the non-infection stone group (61.1 years) was significantly higher than that of patients in the infection stone group (54.4 years) (*P* < 0.05). In addition, the body mass index (BMI) was significantly higher in the non-infection stone group than in the infection stone group (*P* < 0.05). No statistically significant differences were observed between the two groups with respect to sex distribution or blood pressure (*P* > 0.05).

**Table 1:** General characteristics of the patients

**General characteristics**	**Non-infection group (*n* = 23)**	**Infection group (*n* = 70)**	$x$^2^/t	***P-value***
N	23(24.7)	70(75.3)		
Gender			0.012	0.911
Male	5(21.7)	16(22.9)		
Female	18(78.3)	54(77.1)		
Age	61.1 ± 11.6	54.4 ± 11.6	2.431	0.017
Blood pressure				
Systolic blood pressure	128.7 ± 19.0	130.5 ± 16.2	−0.443	0.659
Diastolic blood pressure	75.4 ± 12.5	75.7 ± 12.2	−0.104	0.917
Diabetes [n (%)]	5(21.7)	7(10.0)	2.123	0.145
BMI (kg/m^2^)	28.0 ± 7.1	24.9 ± 4.8	2.425	0.017

### Metabolic profiles of patients in the infection stone and non-infection stone groups

Analysis of 24-h urine metabolic parameters demonstrated that urine volume was significantly lower in the non-infection stone group compared with the infection stone group (*P* < 0.05). No statistically significant differences were identified between the two groups in other metabolic parameters, including urinary calcium, oxalate, citrate, uric acid, sodium, or phosphate excretion (all *P* > 0.05) (Table 2).

**Table 2:** Metabolic profiles of the patients

Metabolic indicators	Group		*P-value*
	**Non-infection group (*n* = 23)**	**Infection group (*n* = 70)**	
Serum biochemistry			
Calcium [M (Q1, Q3), mmol/L]	2.26(2.16,2.34)	2.24(2.17,2.31)	0.656
Magnesium [M (Q1, Q3), mmol/L]	0.86(0.82,0.92)	0.84(0.80,0.89)	0.399
Phosphorus (mmol/L)	1.26 ± 0.16	1.22 ± 0.25	0.567
Chloride (mmol/L)	106.80 ± 2.65	107.50 ± 3.86	0.424
Sodium (mmol/L)	143.00 ± 1.86	142.08 ± 2.63	0.125
Uric acid [M (Q1, Q3), μmol/L]	346.00(287.00,364.00)	366.00(294.00,409.00)	0.157
PTH [M (Q1, Q3), ng/mL]	46.56(33.40,57.17)	52.27(36.14,72.83)	0.264
BUN [M (Q1, Q3), mmol/L]	6.32(4.72,7.83)	5.70(4.26,8.54)	0.961
Creatinine [M (Q1, Q3), μmol/L]	58.60(49.80,77.00)	67.30(59.15,128.85)	0.034
eGFR [M (Q1, Q3), mL/min]	100.51(78.72,107.20)	88.66(42.81,107.77)	0.233
Random urine			
pH [M (Q1, Q3)]	6.50(6.00,7.00)	7.00(6.50, 7.50)	0.016
24-h urine			
Calcium [M (Q1, Q3), mmol/d]	1.52(1.01,3.30)	1.82(1.10,3.22)	0.986
Sodium [M (Q1, Q3), mmol/d]	122.16(104.16,169.40)	136.36(102.30,176.05)	0.341
Potassium (mmol/d)	26.52 ± 9.89	28.29 ± 11.49	0.509
Phosphorus [M (Q1, Q3), mmol/d]	11.14(9.14,14.27)	11.58(7.45,15.77)	0.901
Chloride [M (Q1, Q3), mmol/d]	102.70(88.70,138.15)	104.76(82.50,141.40)	0.682
Uric Acid [M (Q1, Q3), μmol/d]	2551.90(1964.10,3102.70)	2443.00(1738.80,2895.00)	0.302
Volume [M (Q1, Q3), L/d]	1.50(1.25,1.70)	2.10(1.70,2.50)	< 0.001

Data are presented as median (interquartile range) [M (Q1, Q3)] or mean ± standard deviation

*eGFR* estimated glomerular filtration rate, *PTH* Parathyroid hormone, *BUN* Blood urea nitrogen

### Univariate logistic regression analysis

Univariate logistic regression analysis showed that age, BMI, urinary pH, and 24-h urine volume were significantly associated with the formation of upper urinary tract infection stones (*P* < 0.05). In contrast, serum creatinine, diabetes mellitus, and serum uric acid levels were not significantly associated with infection stone formation (Table 3).

**Table 3:** Univariate logistic regression analysis

Metabolic indicator	Group		*P-value*
	**Non-infection group (*n* = 23)**	**Infection group (*n* = 70)**	
Age	61.1 ± 11.6	54.4 ± 11.6	0.017
BMI (kg/m^2^)	28.0 ± 7.1	24.9 ± 4.8	0.018
Serum creatinine (μmol/L)	58.60(49.80,77.00)	67.30(59.15,128.85)	0.032
pH	6.50(6.00,7.00)	7.00(6.50, 7.50)	0.009
24-h urine volume (L/d)	1.50(1.25,1.70)	2.10(1.70,2.50)	< 0.001
Diabetes [n (%)]	5(21.7)	7(10.0)	0.145
Serum uric acid (μmol/L)	346.00(287.00,364.00)	366.00(294.00,409.00)	0.116

### Multivariate logistic regression analysis of independent risk factors for upper urinary tract infection stone formation

To further investigate independent risk factors associated with the formation of upper urinary tract infection stones, a multivariate logistic regression analysis was performed with stone type as the dependent variable. The infection stone group was defined as the positive outcome, whereas the non-infection stone group served as the reference category. Variables entered into the model included age, BMI, serum creatinine, urinary pH, 24-h urine volume, diabetes mellitus, and serum uric acid. The results showed that diabetes mellitus was significantly associated with the formation of infection stones (*P* < 0.05), although the corresponding confidence interval was relatively wide (OR 12.862, 95% CI 1.237–133.709). In contrast, older age, higher BMI, and lower 24-h urine volume were significantly associated with the formation of non-infection stones (*P* < 0.05) (Table 4).

**Table 4:** Multivariate logistic regression analysis of independent risk factors for infection stone formation

Parameter	Regression coefficient	Standard error	Wald	*P-value*	*OR (95% CI)*
Age	−0.098	0.035	8.014	0.005	0.907 (0.847,0.970)
BMI	−0.172	0.065	7.069	0.008	0.842 (0.741,0.956)
Serum creatinine	0.002	0.006	0.191	0.662	1.002 (0.991,1.014)
pH	1.155	0.564	4.200	0.040	3.175 (1.052,9.586)
24-h urine volume	2.233	0.662	11.382	< 0.001	9.331 (2.549,34.151)
Diabetes	2.554	1.195	4.572	0.032	12.862 (1.237,133.709)
Serum uric acid	0.006	0.004	2.467	0.116	1.006 (0.998,1.015)

### Stones composition analysis

A total of 93 stone samples were included in this study. Of these, 70 samples (75.3%) were classified as infection stones, while 23 samples (24.7%) were classified as non-infection stones. Within the infection stone group (*n* = 70), single-component stones were identified in 9 cases (12.9%), with carbonate apatite being the most common single component (8.6%, 6/70). Mixed-component stones were observed in 61 cases (87.1%), among which the combination of struvite and carbonate apatite accounted for the largest proportion (70.0%, 49/70). In the non-infection stone group (*n* = 23), only one type of single-component stone was identified, namely whewellite (calcium oxalate monohydrate), which was also the most prevalent stone type (56.5%, 13/23). Mixed-component stones were detected in 10 cases (43.5%), with the combination of whewellite and carbonate apatite being the most common (26.1%, 6/23) (Table 5).

**Table 5:** Stone composition in infection and non-infection stones

Component	**N**	**%**		**N**	**%**
Single					
MAP	3	4.3	COM	13	56.5
CA	6	8.6			
Mixed					
MAP + CA	49	70.0	COM + CA	6	26.1
CA + COM	2	2.9	COM + COD	1	4.3
MAP + CA + AAU	7	10	COM + CA + COD	3	13.0
MAP + CA + COM	3	4.3			
Total	70	100		23	100

*MAP* Struvite, *CA* Carbonate apatite, *COM* Whewellite (calcium oxalate monohydrate), *COD* Weddellite (calcium oxalate dihydrate), *AAU* Ammonium acid urate

## Discussion

Urinary stone disease is one of the most common conditions in urology, with a steadily increasing global incidence over recent decades [15]. In the United States, the prevalence of kidney stones has increased from 1 in 20 adults in 1994 to 1 in 11 adults in 2010 [16]. A national cross-sectional survey in China reported that approximately 1 in 17 adults is affected by urinary stones [17]. Infection stones, which account for 10% to 15% of all urinary calculi, represent a distinct category closely associated with urinary tract infections. Owing to their rapid growth and high recurrence rate, infection stones are among the most challenging stone types to manage clinically [6, 18]. Although the pathogenesis of infection stones and the role of urease-producing bacteria have been extensively investigated, the metabolic characteristics of patients with *Proteus mirabilis*-associated infection stones remain insufficiently explored. To address this gap, the present study strictly applied predefined inclusion and exclusion criteria and enrolled 93 patients with upper urinary tract stones and urine cultures positive for *Proteus mirabilis*. Our analysis demonstrated that advanced age, higher BMI, and lower 24-h urine volume were significantly associated with non-infection stones, whereas diabetes mellitus was identified as an independent risk factor for infection stones. These findings provide a theoretical basis for the prevention and management of upper urinary tract infection stones caused by *Proteus mirabilis*.

In this study, the incidence of non-infection stones increased with age, which is consistent with previous reports [19, 20]. The formation of non-infection stones is closely related to electrolyte disturbances, abnormalities in uric acid/oxalate metabolism, renal tubular acidosis, metabolic syndrome, and other conditions that are more prevalent in older individuals [19, 20]. Previous studies have shown that the incidence of uric acid stones increases with age, whereas infection stones tend to become less common in older populations [21]. Uric acid stone formation is influenced by multiple factors, including metabolic syndrome, renal insufficiency, obesity, insulin resistance, and diabetes, all of which are more frequent with aging [21, 22]. Persistent urinary acidification, defined as urine pH ≤ 5.5, is a major contributor to uric acid stone formation, and insulin resistance is known to reduce urinary pH. Insulin resistance is a common pathological feature of many age-related diseases [22]. Similarly, Zhang et al. [23] analyzed 1520 cases of urinary stones and found an age-related increase in the incidence of uric acid stones. Calcium oxalate stones, which account for approximately 70%−80% of all urinary calculi, also show an age-related increasing trend in both sexes [24]. In males, the incidence rises until approximately 40 years of age, whereas in females the increase becomes more pronounced at older ages [25]. This pattern may be explained by the higher prevalence of stone-related comorbidities, such as hypertension, diabetes, and obesity, as well as age-related changes in diet and intestinal function [26]. No uric acid stones were identified in the present study, which may be attributable to the relatively small sample size.

Our results also demonstrated a significant inverse association between BMI and infection stones, indicating that higher BMI is more strongly associated with the formation of non-infection stones. Obesity is closely linked to insulin resistance, which plays an important role in kidney stone pathogenesis [27]. Insulin resistance reduces renal ammonia production, thereby decreasing urinary buffering capacity and lowering urine pH, which favors the formation of uric acid and calcium oxalate stones [28]. In addition, obesity is associated with chronic systemic inflammation and oxidative stress, which may promote immune cell infiltration and contribute to stone formation [29]. Previous studies have identified oxalate and uric acid as important risk factors for calcium oxalate stones and have demonstrated a clear linear relationship between BMI and 24-h urinary excretion of these substances, with higher BMI associated with increased excretion levels [30]. Obesity may promote stone formation not only through insulin resistance and lowered urine pH, but also via chronic low-grade inflammation and oxidative stress, which can facilitate crystal adhesion and aggregation.

Our study identified low urine volume as a risk factor for non-infection stones. A survey conducted in the United States indicated that individuals with low urine output face a significantly higher risk of developing urinary stones compared with the general population [31]. The formation of kidney stones, regardless of stone type, is a complex multistep process involving urine supersaturation, crystal nucleation, crystal growth, and aggregation [32]. Although multiple models of stone formation have been proposed, nucleation and crystal growth are considered essential initiating processes across all models [33]. When fluid intake is inadequate and urine volume is reduced, solutes such as calcium oxalate, calcium phosphate, and uric acid become supersaturated, thereby triggering nucleation and crystallization. Several studies have demonstrated that increasing fluid intake and urine volume reduces the supersaturation of calcium oxalate, calcium phosphate, and uric acid, effectively preventing stone formation [34]. Some investigators emphasize that low urine volume, resulting from insufficient fluid intake or excessive fluid loss, is among the most critical risk factors for kidney stone development and recommend maintaining a daily urine output greater than 2 L [35]. Fink et al. [36] further suggested maintaining a urine output exceeding 2.5 L per day, noting that diluted urine reduces solute supersaturation and shortens the retention time of crystalline particles within renal tubules, thereby limiting their contact with adhesion surfaces and reducing stone formation risk. These findings support the role of low urine volume in promoting non-infection stone formation and are consistent with the results of the present study.

Our findings also indicate that patients with concurrent *Proteus mirabilis* infection and diabetes mellitus are more likely to develop infection stones. *Proteus mirabilis* is a Gram-negative bacterium and a common causative pathogen of urinary tract infections, accounting for approximately 1–10% of UTI-related pathogens [37]. Compared with the general population, patients with diabetes exhibit increased susceptibility to infections [38]. This vulnerability may be attributed to several mechanisms. First, hyperglycemia, a defining characteristic of diabetes, increases glucose concentrations in blood and urine. Because glucose serves as an energy source for *Proteus mirabilis*, this metabolic environment supports bacterial proliferation and enhances virulence [39]. Second, immune dysfunction is common in diabetic individuals, resulting in impaired urinary tract defense mechanisms, including deficits in neutrophil adhesion, chemotaxis, phagocytosis, and natural killer cell activity [38, 40]. Third, autonomic neuropathy may reduce bladder sensation and promote urinary retention, leading to stagnant, glucose-rich urine that further facilitates bacterial growth and weakens phagocytic efficacy [41]. In addition, diabetes may induce renal impairment characterized by reduced glomerular filtration rate and tubular dysfunction, thereby altering renal excretory function and increasing the risk of infection stone formation [42, 43]. It should be noted, however, that the number of diabetic patients included in this study was limited, resulting in an unstable odds ratio estimate. The precise magnitude of this association requires confirmation in larger prospective studies.

Previous studies have consistently reported a high prevalence of insulin resistance among patients with diabetes mellitus [44]. Insulin resistance reduces renal ammonia production, leading to decreased urine pH [28], which may disrupt the urinary environment required for infection stone formation. Additionally, diabetic patients experience urinary tract infections more frequently and with greater severity than non-diabetic individuals [45]. In the presence of a high bacterial load of *Proteus mirabilis* with strong urease activity, urine pH may rise rapidly during severe infections. Accordingly, urine pH may exhibit an increasing trend in the context of severe infection. Due to the limited number of diabetic patients included in the present study, this hypothesis could not be adequately evaluated and should be addressed in future investigations. Furthermore, the urinary pH values measured in this study were derived from random urine samples, which are susceptible to various confounding factors, including diet and timing of collection. Nonetheless, the results of our analysis are in alignment with conclusions from prior studies. To minimize potential interference, all patients were instructed to provide urine specimens while in a fasting state. Previous literature has also supported the utility of pH assessment in fasting random urine samples [46]. This limitation should be acknowledged. Future investigations with larger sample sizes, additional metabolic parameters, and repeated measurements are warranted to further reduce the influence of confounding variables.

The findings of this study have important clinical implications for the prevention of both infection and non-infection stones. In clinical practice, particular attention should be given to older individuals, patients with a high BMI, and those with low urine output. Such patients may benefit from increased fluid intake, dietary modification, and body weight control to reduce the risk of non-infection stone recurrence. In patients with urinary tract infections caused by *Proteus mirabilis*, the presence of diabetes mellitus markedly increases the likelihood of infection stone formation. Therefore, strict glycemic control should be emphasized to prevent infection stone development, especially in patients with confirmed *Proteus mirabilis* infection.

Several limitations of this study should be acknowledged. First, this was a single-center retrospective study with a relatively small sample size, which may limit the generalizability of the findings. Future studies should expand the sample size and conduct multicenter prospective investigations to validate these results. Second, the potential influence of confounding factors, such as dietary habits and medication use, could not be fully excluded. These factors may affect stone composition and should be incorporated into future studies to allow a more comprehensive assessment of their impact.

## Data Availability

The datasets used and analyzed during the current study are available from the corresponding author on reasonable request.

## Abbreviations

eGFR: Estimated glomerular filtration rate
BUN: Blood urea nitrogen
PTH: Parathyroid hormone
BMI: Body mass index
MAP: Magnesium ammonium phosphate hexahydrate
CA: Carbonate apatite
COM: Calcium oxalate monohydrate
COD: Calcium oxalate dihydrate
AAU: Ammonium acid urate
